# Remarks on Crito and Dr. Cayley

**Published:** 1807-07

**Authors:** 


### *To the Editors of the Medical and Physical Journal.*

GENTLEMEN,

ON reading Crito's paper in your last number, I was induced to turn again to Dr. Cayley's; having a faint recollection, that Dr. or Mr. Stubbs, for we have him under both appellations, had recommended the application of caustic, for the cure of a supposed abscess, not aneurism; and that seems to have been the fact. Now, judging from Crito's paper only, would not any body imagine, that Stubbs had conceived rightly of the complaint, and had proposed, rationally perhaps, and on some new principle, caustic for its cure. So much then for Crito's candour.

On what ground he undertakes to defend such ignorance and illiberality as Stubbs betrays, we know not. Dr. Cayley could not surely have applied such language to any one regularly educated to, and honourably pursuing, the profession; and the very words indeed of Stubbs, if fairly stated, would proclaim him, without further observation, an empiric.

We are sorry, on the other hand, that Mr. Strother should have been so unnecessarily brought forward; every purpose of Dr. Cayley's recital, at least every purpose that we will allow ourselves to think about, might have been answered without it. If he "politely acquiesced" in the means which Dr. Cayley had it in his mind to pursue, he ought to have been as politely screened from any imputation of wrong. Indeed, at present he has committed no material error; on the contrary, he has corrected his first opinion, and has discovered and announced the disease, before Doctor Cayley's presence was required. And who would not demand a consultation in such a case, to assist him in the operation he might deem necessary, and to give firmness and composure to the mind of a highly timid patient?

We

We shall beg leave to proceed with a few observations and remarks.

This case of aneurism, " though it adds but little to what was already known, deserves to be preserved." But, except in the recovery of his patient, we see no ground of exultation for Dr. Cayley, which, however, he seems to assume. Does he furnish us with any new diagnostic marks ? or does he discover to us any new principle in his method of cure ? On the contrary, does he not reason falsely on more points than one ? Is it accurate, or is it according to the present improved principles of surgery, to suppose, that by a nice apposition of the edges of the wound in an artery, you can bring them to unite again without affecting the calibre of that artery ? If such a thing be practicable, which, in a large artery, such for instance as one of the tibials, we have no conception of, it ought to be set about very early after the accident. While here, in two months at least after the infliction of the wound, Dr. Cayley is applying continued pressure, not strictly under an idea of obliterating the artery, but in the expectation of bringing the wounded parts of it into contact, and of promoting thereby, as he rather unfortunately expresses it, their " cicatrization."

The cicatrization " of these parts," for we see no objection to the term, in the way at least we would consider it, was no doubt accomplished long before Dr. Cayley commenced with his method of cure. And we would now, as soon expect the entire surfaces of a hare-lip to unite, as these; even if you could have them before you in complete adaptation, and in perfect quietude.

The modus operandi of our curative means is an interesting subject of inquiry, and is intimately connected with rational and successful practice. Let us therefore be allowed to inquire, first of all, what is Nature's method of curing an aneurism, if ever she can be said to perform so great a work ? " It consists in a substitutiou of the collateral branches for the arterial trunk." That is, she cannot preserve the diseased or wounded artery, and therefore she gradually obliterates it, and works her operations, through the inosculating branches. Tubes, but just now barely cognizable as such, creeping and clinging to the bones, of no seeming importance in the natural condition of the limb, assume now, a new and important character, and become the means of its salvation ; and from mere threads or capillaries, soon work themselves into large, tortuous, and prominent arteries. This too is precisely the effect of our endeavours, when successful; whether accomplished by the ligature, or by compression simply.

Dr. Cayley, we apprehend, is mistaken in another point. "The blood," says he, "was very readily forced into the artery by gradual pressure." In spurious aneurism of some continuance, we believe this to be impossible. The tumours may be made to recede before the hand, but where is the blood forced to?—Not into the artery again, but into the surrounding cellular substance, or, "into the deeper recesses by the interstices of the muscles;" which might occasion, as the Doctor well observes, "a great deal of pain." But not, we repeat it, by being forced into the artery again, but, "by bursting up the cellular membrane, and the deep connections of the fasciæ."

The cure of aneurism, by compression, is not new in surgery, as Dr. Cayley seems to imagine. Possibly, under some particular circumstances of the case, or condition of the patient, it may become advisable to attempt it in this way; but in the general run of practice it is not likely to be much followed.

This point in surgery seems to be so well settled as to require but little argument now to enforce it. "There are some few instances," says Mr. Sharpe, "of small aneurisms and punctures of the artery from bleeding, doing well by bandage; but they almost all require the operation at last."

Dr. Adams, in the 34th Number of your Journal, has given us an interesting detail of a case so cured. But would any surgeon, on perusing that history, be inclined to pursue a similar mode of treatment, if it could by any means be avoided? Does the simple operation for aneurism bear any comparison to it, in point of anxiety, suffering, or length of time that was required for its completion?

Dr. Cayley, however, was more expeditious in his cure; and Crito's objecting to this being a wound of the anterior tibial artery, because the bleeding seemingly was stopped by the mere dint of the thumb, is preposterous. For what better could have been devised, or what would so soon distinguish an expert surgeon, from a mere pretender in this way, as to see the one boldly advance his finger into the wound to encounter at once the bleeding orifice, while the other would be calling about him for his wonder-working styptics, and would be heaping in the utmost confusion, cloth over cloth, to conceal their want of effect, under a pretence of assisting them in their operation.

To

To lessen too the importance of these arteries, Crito calls them branches merely of the popliteal. The popliteal artery, after giving off many small branches in the ham, is continued, as it were, into the tibials; they cannot in a strict sense be said to branch from it. However this may be, they are important arteries, and are calculated from their situation, in some points of them, to give almost insuperable embarrassment to the surgeon; and we have to regret that Dr. Cayley, in this case, has not given us the precise situation of the aneurism.

Crito, after determining so decidedly against Dr. Cayley's method of cure, and after attributing every thing bad to compression, says, to our surprize, " if a similar case occur in my practice, I will give Dr. Cayley's novel method a fair trial; and if I do not succeed by compression, I will next try Dr. Stubb's." We sincerely wish his patient a safe deliverance.

We have yet to learn indeed, what this Dr. Stubb's method of cure may be; for certainly, in the case we are considering, the caustic was proposed in entire ignorance —not with any reference to aneurism, but to give vent to a tumour, that contained, as he ignorantly said, " a large collection of matter, covered with proud flesh and skin."

There is not we think, and we are sorry to make the observation, any superior intelligence manifested, or any example that we could wish to imitate, in Crito's case of wounded ulnar artery. What does he mean by sewing the artery? Is it giving it the glover's stitch, or is the passing a ligature round it, a species of sewing? In the latter sense, indeed we would agree with him; but neither the one nor the other, could be done with accuracy or effect, without first dilating so small a wound, and slitting up the fascia, under which the artery runs. And if once arrived at this stage of the operation, would any body (in the present state of surgery) hesitate for a moment between obliterating it at once by a single pull of the ligature; and trusting on the other hand, to a nice, but precarious stitching. As Crito declines saying, which of the means ultimately put a stop to the hæmorrhage, we shall not presume to decide for him; we cannot, however, attach much importance to any of those which he has mentioned; and we should be sorry, in such a case, to pronounce the patient in security, having put, according to the words before us, a little lunar caustic into the wound, and secured it only with a pledgit of lint soaked in tincture of Benjamin.

Crito, so far, if he has not much illumined our minds, neither has he much discomposed them; but we now come to a passage full of danger, and merits therefore the severest reprehension. He asks, alluding to the aneurismal tumour, " wherein consisted the difference of laying it open with a lancet or cautic?" and thus replies. " The latter was certainly preferable, if it had the appearance of aneurism."

Now, surely, of all the methods of opening a tumour of a doubtful nature, but particularly if you suspect it to be aneurism, caustic must be the most objectionable. A puncture with a lancet, or with one of Hey's needles, would in such a case, sufficiently ascertain its nature; and a wound so produced would immediately heal again, if it became adviseable to promote it. But far otherwise would it be with an opening produced by caustic; the eschar of which moreover might give way at an unguarded moment, and no effectual assistance perhaps being at hand, the patient might irrecoverably sink in a few pulsations.

" I would take this opportunity," says Mr. Hey, " of strongly recommending the method here used of exploring the contents of tumours in doubtful cases. I have used it upon several occasions with great satisfaction and advantage. There are few doubtful cases in which any harm could be done by the puncture of a couching needle. The contents of the tumour may be generally ascertained by such a puncture, the pain of which is trifling, and the wound is soon healed."—— *Vide Hey's Surgery.*

A PRACTITIONER IN DERBYSHIRE.

*May* 26, 1807.

